# *Kadsura coccinea* Roots Ameliorated Alcohol-Induced Liver Injury by Modulating Oxidative Stress Through the Regulation of the Nrf2/MAPK Signaling Pathway

**DOI:** 10.3390/ijms27125362

**Published:** 2026-06-14

**Authors:** Yashi Wang, Shiqi Liu, Aamer Muhammad, Jiahao Chen, Zhuocheng Xie, Yuxuan Yao, Chuanle Li, Wei Wang, Yupei Yang, Bin Li

**Affiliations:** TCM and Ethnomedicine Innovation & Development International Laboratory, School of Pharmacy, Hunan University of Chinese Medicine, Changsha 410208, China

**Keywords:** alcohol-induced liver injury, *Kadsura coccinea*, oxidative stress, Nrf2, MAPK

## Abstract

The present investigation evaluated the therapeutic potential of ethanol-derived extracts from *Kadsura coccinea* root (KCR) against alcohol-induced liver injury (ALI) utilizing a murine experimental system. Male Balb/c mice were administered alcohol intragastrically in a stepwise manner over 8 weeks to establish the ALI model. Experimental outcomes demonstrated that KCR administration substantially improved hepatic functional status, evidenced by marked reductions in circulating hepatic enzymes, specifically aspartate aminotransferase (AST) and alanine aminotransferase (ALT). KCR also increased glutathione (GSH) activity, reduced malondialdehyde (MDA) levels in the liver, and exerted antioxidant effects by boosting the expression of enzymes such as superoxide dismutase (SOD), catalase (CAT), and glutathione peroxidase 4 (GPX4). Additionally, metabolomic and transcriptomic analyses identified metabolites and pathways closely linked to oxidative stress, including Glutathione metabolism and the MAPK signaling pathway. Further mechanistic studies revealed that KCR could decrease the phosphorylation of p38, JNK, and ERK, while increasing the expression of Nrf2, HO-1, and NQO1. In conclusion, KCR alleviates ALI by modulating the MAPK/Nrf2 pathway, restoring redox homeostasis, enhancing antioxidant defenses, and improving metabolic disorders.

## 1. Introduction

Liver diseases constitute a severe global public health threat, causing over two million annual deaths and accounting for approximately 4% of worldwide mortality. Chronic alcohol consumption is a primary etiological factor for diverse liver disorders, with epidemiological data showing that 43% of the global adult population engages in alcohol drinking, indicating the high prevalence of this risk factor [1]. Without timely and effective clinical intervention, acute alcoholic liver injury (ALI) progresses progressively from simple hepatic steatosis and alcoholic steatohepatitis to irreversible hepatic fibrosis, cirrhosis, and even hepatocellular carcinoma [2]. The pathogenesis of alcoholic liver injury is multifactorial, involving ethanol-induced cytotoxicity, redox imbalance, inflammatory responses, mitochondrial dysfunction, endoplasmic reticulum stress, and hepatic metabolic disorders [3,4,5].

Dysregulated cellular redox homeostasis is recognized as a central driver of alcoholic liver damage. As the predominant organ for ethanol biotransformation, the liver generates abundant reactive oxygen species (ROS) and free radicals during alcohol metabolism. Under physiological conditions, endogenous antioxidant systems maintain cellular redox equilibrium; however, ethanol metabolites impair hepatic antioxidant capacity, leading to excessive ROS accumulation and oxidative injury in hepatocytes [6,7,8]. Excessive ROS further activates the mitogen-activated protein kinase (MAPK) signaling cascade, bridging alcohol exposure and hepatic oxidative stress [9]. Specifically, oxidative stress triggers the dissociation of the Trx-ASK1 complex, thereby activating downstream JNK and p38 MAPK pathways in TNF-α-mediated liver injury [10], while altered p38 MAPK dephosphorylation is also closely associated with metabolic stress during liver damage [11].

The transcription factor nuclear factor E2-related factor 2 (Nrf2) serves as a master regulator of cellular antioxidant defense. This conserved signaling pathway governs the expression of cytoprotective genes responsible for free radical scavenging and xenobiotic detoxification [12]. Upon activation, Nrf2 translocates into the nucleus and binds to antioxidant response elements (AREs), thereby upregulating phase II detoxification enzymes and antioxidant proteins, including HO-1 and NQO1, to enhance cellular stress resistance [13,14]. Accumulating evidence has validated the crucial antioxidant and hepatoprotective roles of Nrf2 activation [15]. Notably, numerous natural botanical products exert hepatoprotective effects via modulating the MAPK/Nrf2 signaling axis [16,17], highlighting the promising clinical potential of natural compounds for ALI treatment.

*Kadsura coccinea* (Lem.) A.C. Smith (Heilaohu), a traditional medicinal plant endemic to southern and southwestern China, has been conventionally used for the clinical management of liver diseases, rheumatism, and malignant tumors [18,19]. Phytochemical studies have demonstrated that lignans and triterpenoids, particularly dibenzocyclooctadiene lignans, are the characteristic and predominant bioactive constituents of *K. coccinea*. Our previous chemical profiling confirmed lignans and triterpenoids as the major components of *Kadsura coccinea* root extracts (KCR) via UPLC-Q-Exactive Orbitrap-MS and UHPLC analysis [20]. Modern pharmacological studies have verified that *K. coccinea*-derived lignans possess antioxidant, hepatoprotective, and anti-fibrotic activities [21]. For instance, acetylbinankadsurin A isolated from KCR exhibits potent anti-fibrotic effects [18]. Our prior study also demonstrated that KCR alleviates oxidative stress and hepatocyte apoptosis, thereby ameliorating acetaminophen-induced acute liver injury [22], which firmly supports its hepatoprotective property. ALI is a common and clinically intractable hepatic disorder with limited effective therapeutic options. Nevertheless, the protective effects and underlying molecular mechanisms of KCR against alcoholic liver injury remain unreported. Accordingly, this study was designed to comprehensively investigate the protective role and molecular mechanism of KCR in alleviating alcoholic liver injury, aiming to provide an experimental basis for its clinical application.

## 2. Results

### 2.1. Phytochemical Profile of KCR

Our research team previously employed ultra-high-performance liquid chromatography coupled with Q-Exactive Orbitrap mass spectrometry (UPLC-Q-Exactive Orbitrap/MS) and identified a total of 54 compounds from *Kadsura coccinea* roots, with triterpenoids and lignans as the predominant chemical constituents. We further summarized the characteristic fragmentation pathways of triterpenoids and lignans in *K. coccinea* roots, which can be used to deduce and identify unknown compounds with similar structural features. In addition, quantitative determination by ultra-high-performance liquid chromatography (UHPLC) showed that the contents of schisandronic acid, heilaohuacid G and seco-coccinic acid F were 0.21%, 2.62% and 2.01%, respectively. These three ingredients were confirmed to be the most abundant characteristic components in *K. coccinea* roots [20]. Moreover, isolated and purified monomeric constituents were analyzed by high-performance liquid chromatography with a diode array detector (HPLC-DAD). A total of 8 compounds were further identified from *Kadsura coccinea* roots, which were also primarily dominated by lignans and triterpenoids [23].

### 2.2. KCR Ameliorated the Pathological State of ALI Mice

**Figure 1:**
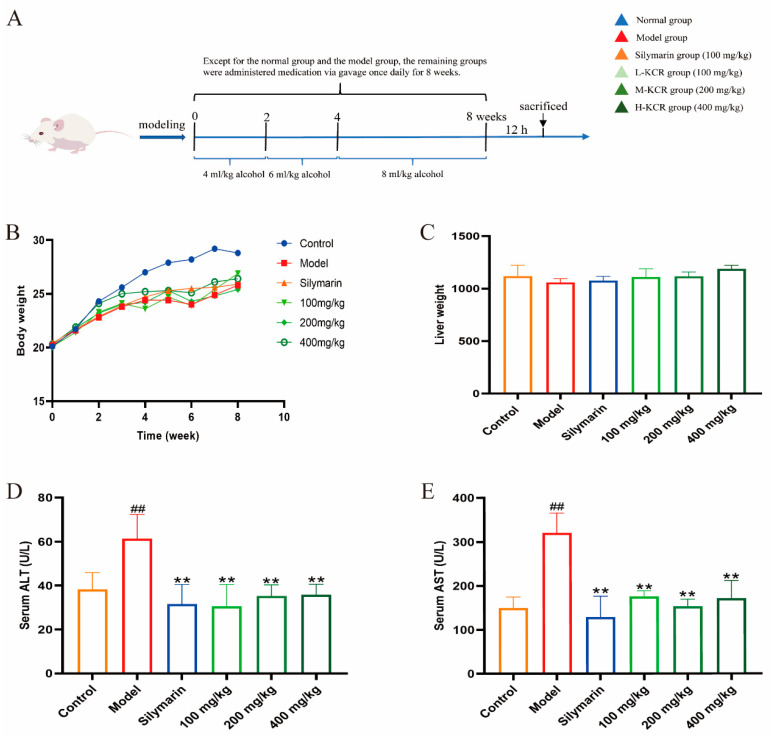
Impact of KCR on pharmacodynamic effects related to ALI in mouse models. (**A**) Diagram of the experimental procedure; (**B**) Weight gain charts for each group; (**C**) Liver weight charts for each group; (**D**,**E**) Serum levels of ALT and AST. Data are shown as the mean ± SEM (n = 6), ## *p* < 0.01 vs. control; ** *p* < 0.01 vs. model.

Designed and conducted animal experiments (Figure 1A) to evaluate the protective effects of KCR against ALI. The results show that the six groups of mice had different rates of weight gain over 8 weeks. The CG gained weight faster than the model and treatment groups, and the treatment groups also had higher body weights than the model group (Figure 1B). However, there were no significant differences in liver weight among the groups (Figure 1C). Serum transaminase activities, comprising alanine and aspartate aminotransferases, serve as sensitive biochemical markers of hepatocellular integrity. The ethanol-exposed model cohort exhibited marked elevation in both enzymatic parameters. KCR administration at all evaluated dosages significantly ameliorated these alcohol-induced elevations (*p* < 0.01).

**Figure 2:**
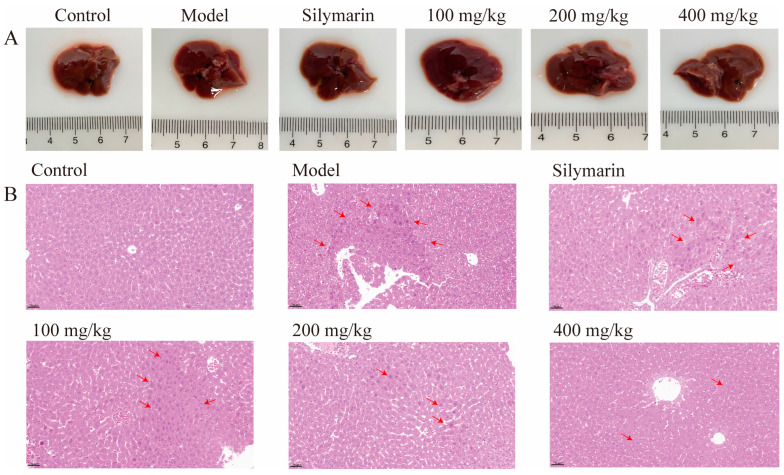
Histopathology effects of KCR on mice with ALI. (**A**) Liver morphology images for each group; (**B**) HE-stained liver sections of the alcohol-induced liver injury model. All images are visualized at 100×. The red arrow indicates inflammatory cell infiltration in the liver tissue and hepatocyte necrosis.

Upon observing the liver morphology across different groups, it was noted that the liver in the MG appeared bright red, which may indicate hemorrhage, necrosis, and inflammatory symptoms. However, as the KCR dose increased, the liver color gradually resembled that of the CG (Figure 2A). HE staining showed that KCR could alleviate liver injury caused by alcohol consumption. Liver sections from MG mice displayed infiltration of inflammatory cells and hepatocyte necrosis within the hepatic tissue. In contrast, KCR treatment significantly mitigated the liver tissue damage (Figure 2B). Additionally, according to the Ishak scoring system (Supporting Information, Table S1), the MG scored 2 for hepatocyte necrosis and 2 for inflammation, with no liver fibrosis detected (Supporting Information, Table S2). After KCR treatment, the liver pathology was alleviated, especially in the high-dose group, which showed the most significant therapeutic effect. This suggests that KCR can alleviate alcohol-induced liver injury.

### 2.3. KCR Can Enhance the Antioxidant Defense Capacity Against ALI

**Figure 3:**
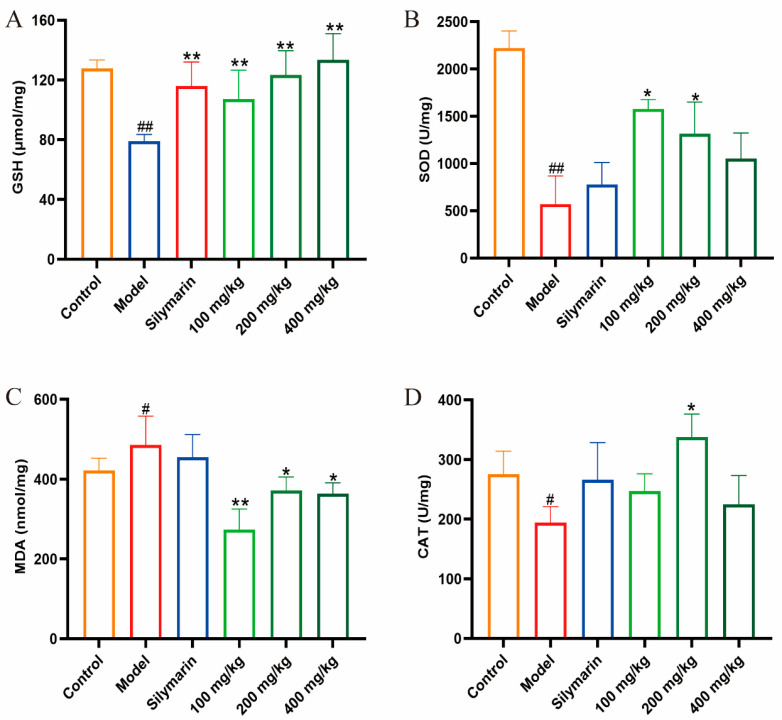
Effects of KCR on oxidative stress-related indicators in the liver. (**A**–**D**) Levels of GSH, SOD, MDA, and CAT in the mouse liver. Data are shown as the mean ± SEM (n = 6), # *p* < 0.05 vs. control; ## *p* < 0.01 vs. control; * *p* < 0.05 vs. model; ** *p* < 0.01 vs. model.

Oxidative cellular damage typically depletes glutathione reserves and superoxide dismutase and catalase activities while elevating malondialdehyde levels, thereby disrupting hepatic redox homeostasis. To assess KCR effects on oxidative stress following ethanol exposure, we examined changes in redox-related parameters across experimental groups. Ethanol-exposed animals exhibited reduced antioxidant capacity, evident from decreased glutathione content, superoxide dismutase and catalase activities, alongside elevated malondialdehyde levels compared to untreated controls. KCR treatment effectively reversed these alcohol-induced redox perturbations (Figure 3).

**Figure 4:**
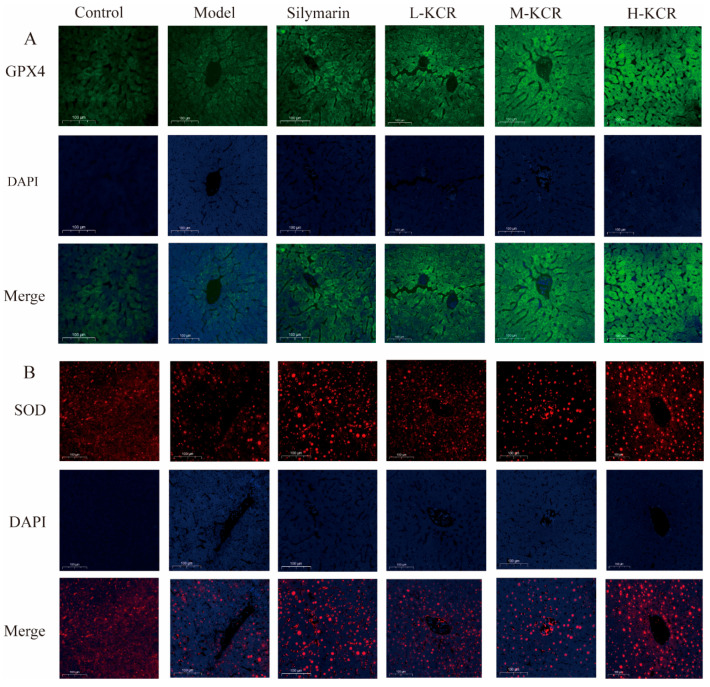
The effect of KCR on antioxidant enzymes in the liver. (**A**,**B**) Immunofluorescence of antioxidant enzymes GPX4 and SOD in mouse liver. All images are visualized at 100×.

GPX4 and superoxide dismutase represent critical antioxidant enzymes maintaining cellular redox balance and protecting against oxidative damage. Immunofluorescence microscopy revealed diminished expression of both enzymes in ethanol-exposed animals, whereas KCR-treated animals displayed enhanced expression (Figure 4). These findings suggest that ethanol consumption induces oxidative stress, while KCR activates antioxidant enzyme expression, thereby protecting against hepatocellular oxidative damage. Collectively, glutathione content, superoxide dismutase, catalase, and malondialdehyde levels, together with GPX4 and SOD immunofluorescence data, demonstrate that KCR significantly enhances hepatic antioxidant defenses against alcoholic injury.

### 2.4. Effects of KCR on Liver Metabolomics in Mice with ALI

**Figure 5:**
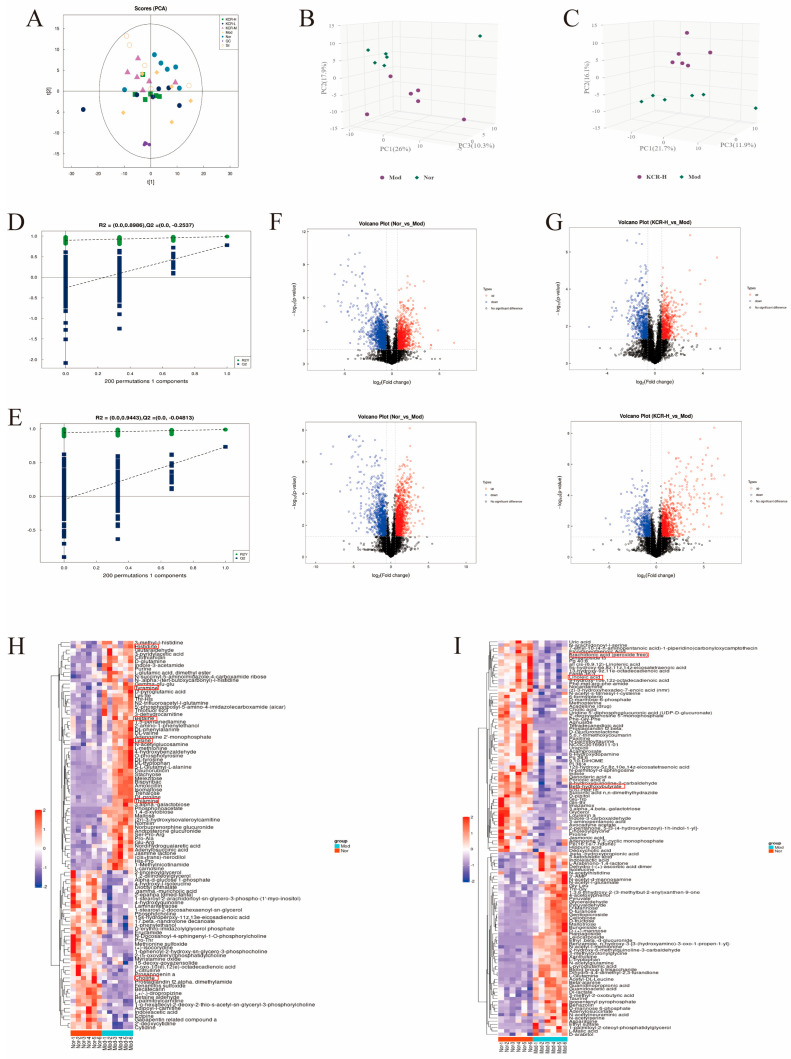
Metabolomics analysis of the KCR intervention. (**A**) PCA distribution plots for each group; (**B**) PCA plot comparing NG and MG; (**C**) PCA plot comparing MG and H-KCR; (**D**) OPLS-DA permutation test plot for NG and MG; (**E**) OPLS-DA permutation test plot for H-KCR and MG; (**F**) Volcano plots comparing NG and MG (top in the positive ion mode, and bottom in the negative ion mode); (**G**) Volcano plots comparing H-KCR group and the MG (top in the positive ion mode, and bottom in the negative ion mode); (**H**,**I**) Heatmaps of enriched differential metabolites between NG and MG (positive ion mode on the left, negative ion mode on the right).

To investigate the overall changes in the liver across different experimental groups, multivariate statistical analysis was performed on liver metabolomics datasets. Clear separation trends among the metabolic profiles of all groups were observed in principal component analysis (PCA) score plots (Figure 5A). Focused comparative analysis revealed significant metabolic divergence between the NG and MG groups, as well as between the MG and H-KCR groups, with clear segregation patterns visualized in PCA plots (Figure 5B,C). The partial least squares-discriminant analysis (PLS-DA) score plots also demonstrated strong separation among the H-KCR, NG, and MG groups (Figure 5D,E). The robustness of the PLS-DA model was validated through response permutation testing, with both Q2 and R2 values above 0.5, indicating the model’s adequacy. Differential metabolites were identified based on variable importance in projection (VIP) scores derived from the PLS-DA model combined with Student’s *t*-test, where VIP scores and *p*-values provided different levels of statistical significance. The screening thresholds for significantly altered metabolites were set at *p* < 0.05 and VIP > 1.0.

**Figure 6:**
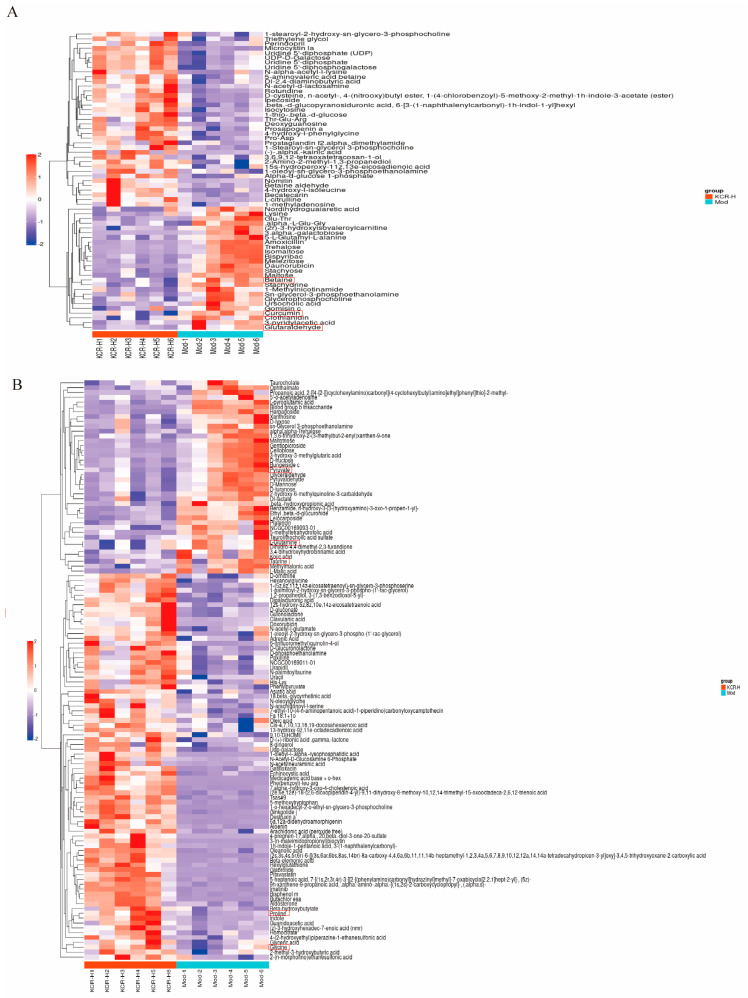
Metabolomics analysis of the KCR intervention. (**A**,**B**) Heatmaps of enriched differential metabolites between the H-KCR group and MG (positive ion mode on the left, negative ion mode on the right).

In positive ion mode, there were 940 up-regulated and 1263 down-regulated differential metabolites between the NG and MG (Figure 5F). Heatmap enrichment analysis of metabolites revealed key compounds such as histidine, lysine, tyramine, betaine, thiamine, and choline (Figure 5H). Between the high-dose group and the MG, there were 621 up-regulated and 651 down-regulated differential metabolites (Figure 5G). Heatmap enrichment analysis of metabolites identified key pathway-related compounds, including curcumin, glutaraldehyde, and betaine (Figure 6A).

In the negative ion mode, there were 1369 up-regulated differential metabolites and 959 down-regulated differential metabolites between the NG and the MG (Figure 5F). Heatmap enrichment analysis of metabolites identified key metabolites such as linoleic acid, arachidonic acid (peroxide-free), and β-hydroxybutyrate (Figure 5I). Between the high-dose group and the MG, there were 928 up-regulated differential metabolites and 653 down-regulated differential metabolites (Figure 5G). Heatmap enrichment analysis identified key pathway-related metabolites like pyruvate, L-glutamine, taurine, proline, and glycine (Figure 6B).

**Figure 7:**
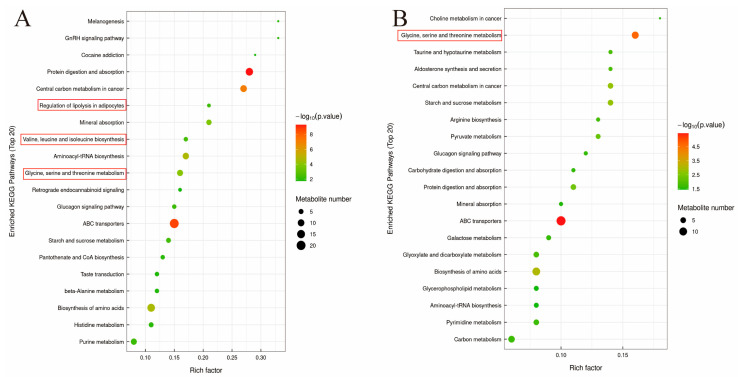
Metabolomics analysis of the KCR intervention. (**A**,**B**) KEGG pathway enrichment plots for comparing NG and MG, and H-KCR group and MG.

Further KEGG enrichment analysis revealed that the key metabolic pathways between the NG and the MG were regulation of lipolysis in adipocytes, valine, leucine, and isoleucine biosynthesis, and glycine, serine, and threonine metabolism (Figure 7A). The key metabolic pathways between the high-dose group and the MG were aldosterone synthesis and secretion, biosynthesis of amino acids, and pyruvate metabolism (Figure 7B).

### 2.5. Transcriptomic Analysis of the KCR Intervention

**Figure 8:**
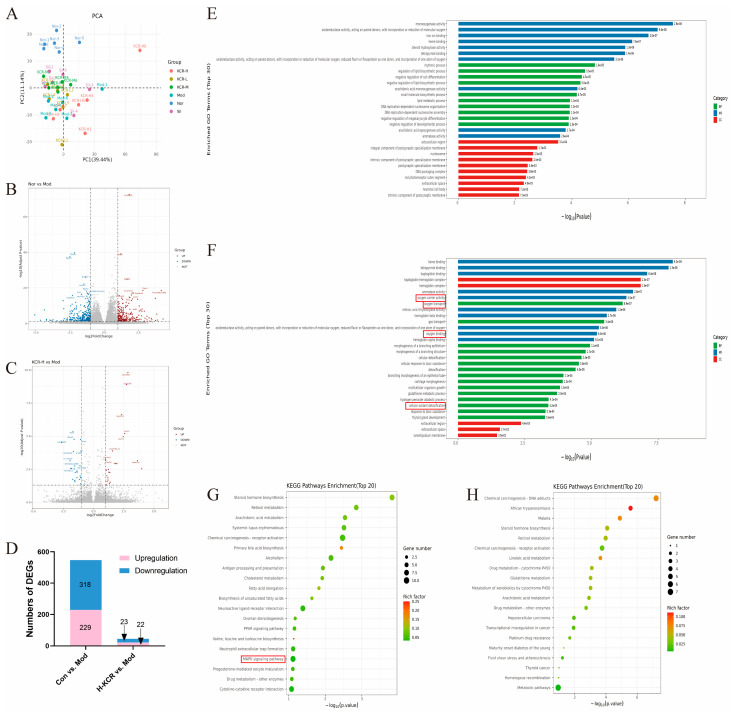
Transcriptomic analysis of the KCR intervention. (**A**) PCA plots for each group; (**B**,**C**) Volcano plots comparing the normal and model groups, and comparing the model and H-KCR groups, with red indicating upregulated genes and blue indicating downregulated genes; (**D**) The number of differentially expressed genes in the two comparisons, with pink representing upregulated genes and light blue representing downregulated genes; (**E**,**F**) GO analysis plots for comparisons between the normal and model groups, and between the model and H-KCR groups; (**G**,**H**) KEGG pathway analysis plots for the comparisons between the normal and model groups, and between the model and H-KCR groups.

To evaluate the potential mechanisms of KCR in treating ALI, transcriptomic analysis was performed using samples from six groups: normal, model, positive control, L-KCR, M-KCR, and H-KCR. PCA revealed clear separation among the model, control, and H-KCR groups, indicating significant gene expression changes (Figure 8A). Next, the study focused on transcriptional changes in these three groups. Differential expression analysis was conducted with the criteria of *p* < 0.05 and |log2FC| > 1 to identify DEGs. The results, shown in stacked bar charts (Figure 8D) and volcano plots, demonstrated that a total of 547 DEGs were identified between the normal and model groups, consisting of 229 upregulated and 318 down-regulated genes (Figure 8B). In the comparison between the model and high-dose KCR groups, 45 DEGs were detected, including 22 upregulated and 23 downregulated genes (Figure 8C).

After identifying the DEGs, the study was conducted using KEGG and GO annotations to elucidate their basic functions. The GO annotation analysis revealed that the functions of DEGs were associated with oxidative stress. Most of the significantly enriched GO terms among the identified DEGs were linked to oxidative stress processes and cellular functions (Figure 8E,F). In the top 30 key GO terms comparing the model and high-dose groups, terms such as Oxygen Carrier Activity, Oxygen Transport, Oxygen Binding, and Cellular Oxidant Detoxification were closely linked to oxidative stress processes. These processes are essential for maintaining normal cellular functions and responding to oxidative stress.

KEGG enrichment analysis showed that gene alterations between the normal and model groups were associated with pathways such as Steroid hormone biosynthesis, Arachidonic acid metabolism, Cholesterol metabolism, Drug metabolism-other enzymes, MAPK signaling pathway, and Primary bile acid biosynthesis (Figure 8G). In contrast, the gene changes between the H-KCR and model groups were primarily related to pathways including Linoleic acid metabolism, Drug metabolism-cytochrome P450, Drug metabolism-other enzymes, Glutathione metabolism, and Metabolic pathways (Figure 8H).

### 2.6. KCR Suppresses MAPK Pathway Activation

**Figure 9:**
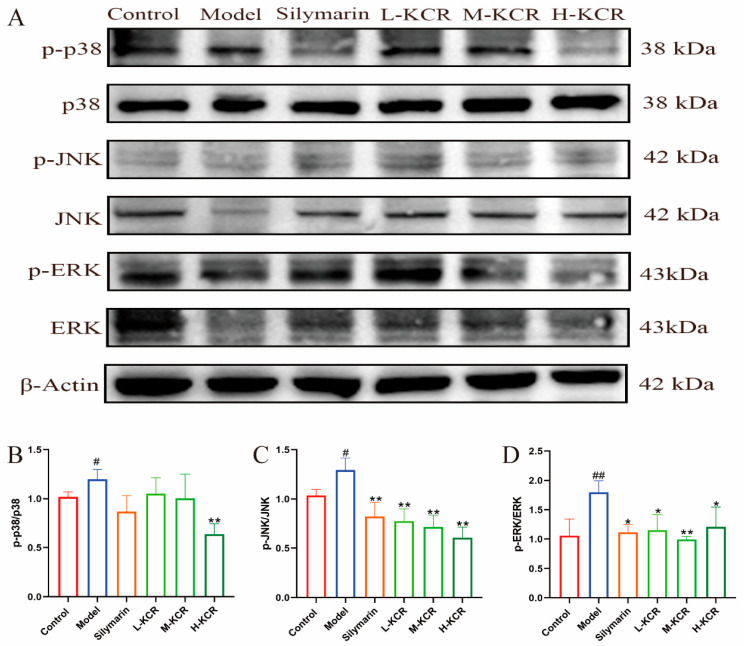
Effects of KCR on hepatic MAPK pathway–related protein levels in mice with ALI. (**A**) Representative WB images for p-p38, p38, p-JNK, JNK, p-ERK, and ERK. (**B**–**D**) Quantitative immunoblot analyses of p38 vs. p-p38, JNK vs. p-JNK, and ERK vs. p-ERK. Data are shown as the mean ± SEM, # *p* < 0.05 vs. control; ## *p* < 0.01 vs. control; * *p* < 0.05 vs. model; ** *p* < 0.01 vs. model.

Ethanol and ethanol-induced ROS overproduction activate MAPK cascades, which serve as critical signal transduction pathways transmitting information from the cell membrane to the nucleus. These pathways regulate diverse cellular processes including inflammatory responses, programmed cell death, and oxidative stress reactions. Transcriptomic KEGG analysis suggested KCR might ameliorate ALI through MAPK pathway modulation. To verify whether KCR participates in MAPK activation to counter ethanol-induced oxidative stress, we examined MAPK kinase activation (p38/JNK/ERK) using immunoblotting (Figure 9A). Compared to control animals, ethanol-exposed animals displayed significantly elevated phosphorylation levels of p38, JNK, and ERK. These phosphorylation events were markedly reduced following KCR intervention (Figure 9B–D). Therefore, within the MAPK signaling network, KCR appears to alleviate alcohol-induced oxidative hepatic injury by suppressing activation of these protein kinases.

### 2.7. KCR Modulates Nrf2 Pathway to Alleviate Hepatic Injury

**Figure 10:**
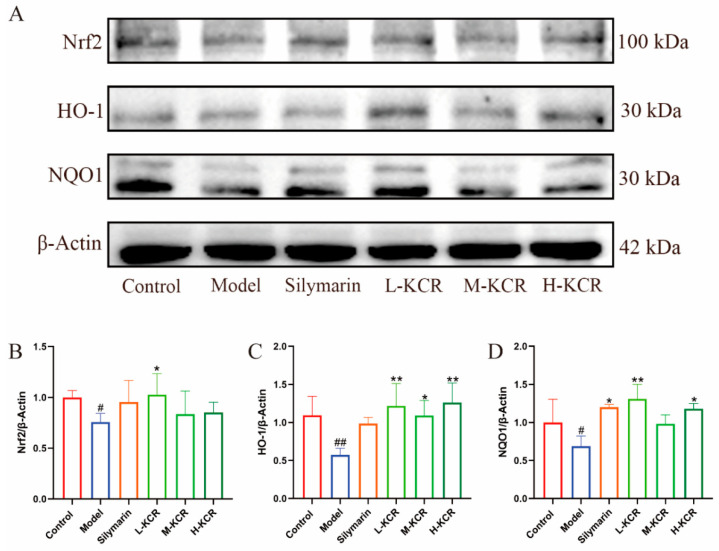
Effects of KCR on hepatic Nrf2 pathway-related protein levels in mice with ALI. (**A**) Representative WB images for Nrf2, HO-1, and NQO1. (**B**–**D**) Quantitative densitometric analysis of Nrf2, HO-1, and NQO1 protein expression. Data are shown as the mean ± SEM, # *p* < 0.05 vs. control; ## *p* < 0.01 vs. control; * *p* < 0.05 vs. model; ** *p* < 0.01 vs. model.

Transcriptomic and metabolomic analyses identified specific metabolites and metabolic pathways closely associated with oxidative stress responses. These findings suggest that ethanol consumption induces oxidative cellular stress, ultimately precipitating hepatocellular damage. The Nrf2-mediated signaling cascade plays a pivotal role in cellular defense against oxidative insults. To investigate whether KCR modulates Nrf2 signaling to ameliorate ethanol-induced oxidative stress, we performed immunoblotting to quantify Nrf2 pathway-related proteins (Figure 10A). In the ALI model, hepatic protein levels of Nrf2 and its downstream antioxidant targets, HO-1 and NQO1, were significantly reduced in ethanol-exposed animals compared to controls. Following KCR intervention, hepatic expression of Nrf2, HO-1, and NQO1 increased relative to model animals (Figure 10B–D). These findings indicate that KCR mitigates alcohol-induced hepatic injury through modulation of the Nrf2-mediated antioxidant response pathway.

## 3. Discussion

Ethanol represents among the most prevalent psychoactive substances consumed globally. Despite its cultural and social acceptance, chronic ethanol exposure poses substantial health risks, particularly to hepatic structure and function, through diverse pathogenic mechanisms. Alcohol-related diseases are increasingly becoming a significant public health concern [1]. When large amounts of alcohol are consumed in a short period, the liver cannot metabolize it quickly, leading to the production of ROS through a series of biochemical reactions. This process causes oxidative damage and triggers ALI [2]. If untreated, it can gradually develop into liver fibrosis, cirrhosis, and liver cancer, severely threatening human health [24]. Currently, there are no effective clinical drugs for ALI. Therefore, developing therapeutic drugs or finding treatment methods is urgent. Effective prevention and treatment of ALI are essential for early detection of various liver diseases. Modern research shows that lignans rich in *K. coccinea* have better hepatoprotective effects and can resist acute liver injury caused by acetaminophen [18,22]. Thus, the present study conducted a series of experiments to explore the mechanism of action of KCR on ALI. In vivo experiments showed that KCR could reduce serum ALT and AST levels and improve liver tissue damage (Figure 1 and Figure 2), indicating that KCR can alleviate liver injury caused by alcohol consumption.

Cellular redox imbalance constitutes a unifying pathogenic mechanism across diverse etiologies of hepatic injury. Whether initiated by ethanol, pharmaceutical compounds, viral pathogens, environmental toxicants, or nutritional factors, oxidative stress represents a common terminal pathway. Chronic hepatic pathologies consistently demonstrate elevated oxidative burden markers, reflecting fundamental disturbances in pro-oxidant and antioxidant equilibrium [25]. Glutathione peroxidase (GPx) is a major selenium-containing protein that maintains the intracellular redox state [26]. GSH, SOD, and CAT are essential antioxidant systems in the human body. SOD produces hydrogen peroxide, which catalase decomposes into water and oxygen and is detoxified by the synergistic action of GSH and GPx. During ALI, ROS are scavenged by antioxidant enzymes such as SOD and CAT, as well as antioxidant substances like GSH, working synergistically with GPx [27]. In this study, in the liver of an alcohol-consuming mouse model, levels of GSH, SOD, and CAT decreased, while MDA levels increased. Simultaneously, the expression of antioxidant enzymes GPX4 and SOD was inhibited. After KCR intervention, the levels of GSH, SOD, and CAT increased, and the level of MDA decreased. Additionally, the expression of antioxidant enzymes GPX4 and SOD was enhanced (Figure 3 and Figure 4). These results suggest that alcohol intake triggers oxidative stress, leading to liver injury. However, KCR intervention enhances the liver’s antioxidant defense system.

Metabolomics and transcriptomics, as core tools in systems biology, have shown powerful applications across various research fields by analyzing gene expression and metabolic end-products [28,29]. In modern research, many scientists have employed metabolomics and transcriptomics to investigate the mechanisms underlying liver diseases. For example, Wang et al. examined how the phenolic-rich extract of *Rhodomyrtus tomentosa* fruits improves non-alcoholic fatty liver by combining transcriptomics and metabolomics [30]. Qin et al. explored the potential mechanism of the polyphenol extract of *Rubus* fruits in alleviating non-alcoholic fatty liver through the combination of metabolomics and transcriptomics analysis [31]. This study also combined metabolomics and transcriptomics to analyze changes in liver metabolites and genes. Metabolomics results indicated the differences in metabolites between the NG and MG groups, as well as between the MG and high-dose groups (Figure 5, Figure 6 and Figure 7). Metabolomic heat-map analyses revealed that curcumin [32] and betaine [33] are strongly associated with the MAPK and Nrf2 signaling pathways, while histidine [34] and lysine [35] are closely linked to oxidative stress responses. KEGG pathway enrichment analysis showed that glycine, serine, and threonine metabolism are closely related to the antioxidant GSH [36], and pathways such as the regulation of lipolysis in adipocytes [37] are also involved in the oxidative stress process. These results suggest that alcohol consumption may disrupt metabolic pathways related to oxidative stress, and KCR can modulate these pathways to alleviate alcohol-induced liver injury.

Subsequently, this study employed transcriptomics to evaluate the potential mechanism by which KCR alleviates ALI. The results showed that 547 DEGs were identified between the NG and MG groups, and 45 DEGs between the model group and the high-dose KCR group. GO enrichment analysis revealed terms such as “Oxygen Carrier Activity,” “Oxygen Transport,” “Oxygen Binding,” and “Cellular Oxidant Detoxification,” which are closely related to the oxidative stress process. These processes are vital for maintaining normal cell function and managing oxidative stress. KEGG enrichment analysis identified two pathways closely associated with oxidative stress: Glutathione metabolism and the MAPK signaling pathway (Figure 8). Excessive ROS can activate the MAPK pathway, and this activation persists even after initial oxidative stress subsides because MAPK activation leads to an increase in ROS production. Increasingly, researchers are investigating mechanisms of liver diseases from the perspective of the MAPK pathway. For example, Tao et al. discovered that ceramide kinase inhibits ferroptosis via the p38 MAPK-HSPB1 pathway, protecting against alcohol-related liver disease [38]; Cui et al. investigated the preventive and therapeutic effects of *Gentiana* extract on alcoholic liver disease through the MAPK/JNK/p38 pathway [39]; Wang et al. found that glabridin alleviates alcohol-induced liver injury by reducing oxidative stress and inflammation through the p38 MAPK/Nrf2/NF-κB signaling pathway [40]. Therefore, this study also evaluated whether KCR affects the MAPK pathway to alleviate ALI. The results showed that compared with the control group, phosphorylation levels of p38, JNK, and ERK were significantly increased in the MG, and these levels decreased markedly after KCR intervention (Figure 9), suggesting that KCR may alleviate ALI by modulating the MAPK pathway.

Nrf2 plays a key role in the regulation of antioxidant responses. A deficiency of Nrf2 leads to increased susceptibility to oxidative damage [41]. When cells are stimulated by factors such as antioxidants and external substances, Nrf2 becomes activated, translocates into the nucleus, forms a complex with MAF proteins, binds to the ARE, and controls the expression of ARE-mediated antioxidant enzyme genes, such as HO-1 and NQO1 [42]. In modern research, some natural products protect ALI by activating the Nrf2 pathway [43]. Additionally, compared to normal mice, the mortality rate of Nrf2-deficient mice fed ethanol increases [44], indicating that the Nrf2/HO-1 signaling axis might be a promising target for ALI treatment [45]. In this study, KCR activated the Nrf2 pathway in vivo (Figure 10), suggesting that KCR may exert its antioxidant effects through the Nrf2 pathway to reduce ALI.

## 4. Materials and Methods

### 4.1. Reagents

Fifty-six percent alcohol by volume (Red Star Erguotou) was acquired from Beijing Red Star Co., Ltd. (Beijing, China). Silymarin reference standard was sourced from Sigma-Aldrich (Merck KGaA, Darmstadt, Germany). Aspartate and alanine aminotransferase assay systems were obtained from Mindray Bio-Medical Electronics (Shenzhen, China). Glutathione, malondialdehyde, superoxide dismutase, and catalase evaluation kits were provided by Nanjing Jiancheng Bioengineering Institute (Nanjing, China). TRIzol reagent was purchased from Invitrogen (Carlsbad, CA, USA). Pre-cast polyacrylamide gels (10% and 12%) were acquired from Wansheng Haotian Biotechnology (Shanghai, China). Enhanced chemiluminescence detection reagents were obtained from Ningbo Youcheng Biomedical Technology (Ningbo, China). Bicinchoninic acid protein quantification kits were sourced from Elabscience (Shenzhen, China). Primary antibodies targeting Nrf2 and mRNA sequencing library preparation kits were acquired from ABclonal Technology (Wuhan, China). Antibodies against HO-1, Keap1, NQO1, JNK, p-JNK, p38, p-p38 MAPK, ERK, and p-ERK were procured from Proteintech (Wuhan, China). Bovine serum albumin, SOD antibodies, GPX4 antibodies, and DAPI were provided by Wuhan Servicebio Technology (Wuhan, China).

### 4.2. Sample Preparation

The dried roots of *Kadsura coccinea* (50 kg) were initially macerated in ethanol for 1 h, followed by sequential reflux extraction with 5 and 4 vol. of 80% ethanol for 2 h each. The extract was filtered, and the filtrate was concentrated under reduced pressure at 60 °C to obtain the ethanol extract (1.3 kg), which was stored at room temperature in a sealed container.

### 4.3. Animal Experimentation

Thirty-six specific-pathogen-free adult male Balb/c mice, weighing 18–20 g, were procured from Hunan Silek Jingda Laboratory Animal Co., Ltd. (Hunan, China) (Quality Certificate No. 430727221101937265). This animal experiment was performed in accordance with the ARRIVE Guidelines 2.0.

Animals were randomly allocated into six experimental cohorts (n = 6 per cohort): (1) vehicle-treated control animals; (2) ethanol-exposed model animals; (3) reference animals receiving silymarin (100 mg/kg body weight); and KCR-treated animals at (4) low-dose (100 mg/kg), (5) medium-dose (200 mg/kg), and (6) high-dose (400 mg/kg) levels. With the exception of the control cohort, all experimental groups received oral administration of 56% ethanol (Red Star Erguotou) according to an escalating dosing protocol: 4 mL/kg during weeks 1–2, increased to 6 mL/kg during weeks 3–4, and escalated to 8 mL/kg for the final four weeks, comprising an eight-week ethanol challenge period. Concurrently, all groups except control and model cohorts received their respective treatments via oral gavage once daily throughout the eight-week period. Following final administration, animals were fasted for twelve hours prior to tissue collection.

### 4.4. Serum Biochemical Parameters

The retro-orbital venous plexus was used for sampling using standard glass capillaries. Following collection, blood specimens were subjected to centrifugal separation to obtain serum fractions. Then, the serum samples were tested for AST and ALT using commercially available diagnostic kits.

### 4.5. Measurement of Hepatic GSH, SOD, MDA, and CAT Levels

Liver specimens were weighed and homogenized in nine volumes of ice-cold extraction buffer. Homogenates were centrifuged at 12,000 rpm for fifteen minutes at 4 °C. Supernatants were collected for preparation of ten percent tissue homogenates. Glutathione content, superoxide dismutase activity, malondialdehyde levels, and catalase activity were determined using commercial colorimetric assay kits following provided protocols.

### 4.6. Hematoxylin and Eosin (HE) Staining

Tissue specimens were fixed in four percent paraformaldehyde solution. Following fixation, samples underwent sequential dehydration, clearing, and paraffin embedding. After dewaxing and rehydration, five-micrometer sections were stained with hematoxylin for three to five minutes, washed, differentiated, and blued. Sections were subsequently stained with eosin for one to two minutes, dehydrated through a graded ethanol series, cleared in xylene, and mounted with neutral synthetic resin. Stained sections were examined and imaged using optical microscopy (China, Servicebio, SWE-CX63, Wuhan, China).

### 4.7. Immunofluorescence Analyses

For immunofluorescence staining, liver sections were blocked with bovine serum albumin at ambient temperature for thirty minutes, then incubated overnight at 4 °C with primary antibodies against SOD and GPX4. Following primary antibody incubation, sections were exposed to Alexa Fluor-conjugated secondary antibodies (sheep anti-rabbit) at ambient temperature for fifty minutes, then counterstained with DAPI for ten minutes protected from light.

### 4.8. Untargeted Metabolomics

Specimens were gradually thawed at 4 °C. Approximately fifty milligrams of each sample was added to pre-cooled methanol/acetonitrile/water solution (2:2:1, *v*/*v*/*v*), vortexed, and subjected to low-temperature ultrasonication for thirty minutes. Extracts were maintained at −20 °C for ten min, then centrifuged at 14,000× *g* for twenty min at 4 °C. Supernatants were collected and dried under vacuum. Untargeted metabolomic profiling was performed via ultra-high-performance liquid chromatography coupled with Q Exactive Orbitrap mass spectrometry (UHPLC-Q Exactive MS) (Thermo Fisher Scientific, Bremen, Germany). Chromatographic separation was achieved using an ACQUITY UPLC HSS T3 column (100 mm × 2.1 mm, 1.8 µm, Waters Corporation, Milford, MA, USA). The mobile phase consisted of solvent A (5 mmol/L ammonium acetate plus 5 mmol/L acetic acid in ultrapure water) and solvent B (acetonitrile). The gradient elution program was performed as follows: 0–1 min, 1% B; 1–9 min, 1–99% B; 9–9.1 min, 99–1% B; 9.1–12 min, 1% B. The flow rate was set at 0.35 mL/min, the column temperature was maintained at 40 °C, and the injection volume was 2 μL. Raw data were converted to mzXML format using ProteoWizard (https://proteowizard.sourceforge.io/, accessed on 1 June 2026). Metabolite identification was performed with an in-house R package based on xcms (version 4.7.3) and CAMERA (version 1.54.0) running on R 4.2.3, and annotated against the Human Metabolome Database (https://hmdb.ca/, accessed on 1 June 2026). Pathway enrichment analysis was conducted using KEGG (https://www.kegg.jp/, accessed on 1 June 2026). Statistical significance was defined as *p* < 0.05.

### 4.9. Total RNA Isolation and Transcriptome Analysis

Total RNA was extracted from hepatic tissue using TRIzol reagent following manufacturer protocols. RNA integrity was assessed by A260/A280 absorbance ratios using a Nanodrop ND-2000 spectrophotometer (Thermo Fisher Scientific, Wilmington, DE, USA), and RNA integrity numbers were determined using an Agilent Bioanalyzer 4150 system (Agilent Technologies, Santa Clara, CA, USA). Only samples meeting quality criteria were utilized for library construction.

Strand-specific paired-end libraries were prepared using ABclonal mRNA-seq Library Preparation Kits (ABclonal Technology Co., Ltd., Woburn, MA, USA). Briefly, mRNA was purified from one microgram total RNA using oligo (dT) magnetic beads, followed by fragmentation with divalent cations at elevated temperatures. First-strand cDNA was synthesized using random hexamer primers and reverse transcriptase. Second-strand cDNA was synthesized using DNA polymerase I, RNase H, and dNTPs. Double-stranded cDNA was adapter-ligated and PCR-amplified. PCR products were purified using AMPure XP beads (Beckman Coulter, Brea, CA, USA). Library quality was assessed on an Agilent Bioanalyzer 4150. Libraries were sequenced on an Illumina NovaSeq 6000 platform (Illumina, Inc., San Diego, CA, USA), generating 150 bp paired-end reads.

Raw reads in FASTQ format were processed using in-house Perl scripts (version 5.30.0.). Adapter sequences were removed, low-quality reads were filtered (bases with quality score ≤ 25 exceeding 60% of read length), and reads containing more than 5% ambiguous bases (N) were discarded. Clean reads were aligned to the reference genome using HISAT2 (version 2.2.1) in strand-specific mode. FeatureCounts (Subread package v2.0.4) was used to quantify reads mapped to each gene. FPKM values were calculated based on gene length and mapped read counts. Differential expression analysis was performed using DESeq2 (version 1.46.0); genes with log_2_ fold change > 1 and adjusted *p* < 0.05 were considered significantly differentially expressed. Gene Ontology and KEGG pathway enrichment analyses were conducted using clusterProfiler (version 4.6.2). Enrichment was considered statistically significant at *p* < 0.05.

### 4.10. Western Blot Analysis

Hepatic tissue proteins were isolated using standard extraction protocols and quantified employing BCA methodology. Protein lysates were resolved through sodium dodecyl sulfate-polyacrylamide gel electrophoresis under denaturing conditions, with electrophoretic separation conducted at 80 V for the initial thirty min, followed by a voltage increase to 120 V for ninety min. Separated proteins were subsequently electrotransferred onto polyvinylidene difluoride membranes. Following transfer, membranes were blocked with five percent non-fat milk solution to prevent non-specific binding. Immunodetection was performed using primary antibodies specific for Nrf2 (dilution 1:1000), HO-1 (1:5000), NQO1 (1:5000), total JNK (1:16,000), p-JNK (1:1000), total p38 (1:10,000), p-p38 (1:1000), ERK (1:10,000), p-ERK (1:1000), and β-actin (1:10,000). Following overnight primary antibody incubation at four degrees Celsius, membranes were exposed to appropriate horseradish peroxidase-conjugated secondary antibodies (1:10,000 dilution) for two hours at ambient temperature.

### 4.11. Statistical Analysis

Results are expressed as arithmetic means with corresponding standard deviations. Statistical comparisons were performed using one-way analysis of variance followed by appropriate post hoc tests. Analyses were conducted using GraphPad Prism version 9.0 (GraphPad Software, La Jolla, CA, USA). Probability values below 0.05 were considered statistically significant.

## 5. Conclusions

This study employed experiments, non-targeted metabolomics, and transcriptomics to investigate KCR effects on ALI. The results showed that KCR can inhibit the reduction of antioxidant enzymes (SOD, CAT, and GPX4) and antioxidant substances (GSH) caused by alcohol intake. Untargeted metabolomics analysis indicated that KCR could modulate ALI by regulating metabolic pathways associated with oxidative stress. In further mechanistic studies, transcriptomics analysis revealed that KCR could modulate the MAPK signaling pathway. It significantly reduced the phosphorylation levels of p38, JNK, and ERK to alleviate ALI. Meanwhile, KCR exerted its antioxidant capacity through the Nrf2 pathway to mitigate ALI. Current research mainly focuses on the overall efficacy of KCR and its correlations with signaling pathways. However, the complexity of KCR’s components has limited in-depth mechanistic research. In the future, we will conduct detailed studies in combination with targeted isolation.

## Data Availability

The original contributions presented in this study are included in the article and Supplementary Materials. Further inquiries can be directed to the corresponding authors.

## Supplementary Materials

The following supporting information can be downloaded at: https://www.mdpi.com/article/10.3390/ijms27125362/s1.

## Abbreviations

ALI	Alcohol-induced liver injury
ALT	Alanine aminotransferase
ART	Antioxidant response element
AST	Aspartate aminotransferase
CAT	Catalase
DEGs	Differentially expressed genes
GO	Gene ontology
GPx	Glutathione peroxidase
GPX4	Glutathione peroxidase 4
GSH	Glutathione
HE	Hematoxylin–eosin
KCR	*Kadsura coccinea* root
KEGG	Kyoto encyclopedia of genes and genomes
MAPK	Mitogen-activated protein kinase
MDA	Malondialdehyde
MG	Model group
NG	Normal group
Nrf2	Nuclear factor E2-related factor 2
PCA	Principal component analysis
PLS-DA	Partial least squares discriminant analysis
ROS	Reactive oxygen species
*SOD*	Superoxide dismutase
VIP	Variable importance in projection
WB	Western blot
