# Supplementary material for: Kadsura coccinea Roots Ameliorated Alcohol-Induced Liver Injury by Modulating Oxidative Stress Through the Regulation of the Nrf2/MAPK Signaling Pathway
Source: Int J Mol Sci. 2026 Jun 14;27(12):5362. doi: 10.3390/ijms27125362 (PMC13299493; doi:10.3390/ijms27125362)
Supplement: Supplementary file 1 [file ijms-27-05362-s001.zip › ijms-4341394-supplementary.pdf]

Kadsura coccinea Roots Ameliorated Alcohol-Induced Liver Injury by Modulating Oxidative Stress Through the Regulation of the Nrf2/MAPK Signaling Pathway

Yashi Wang, Shiqi Liu, Aamer Muhammad, Jiahao Chen, Zhuocheng Xie, Yuxuan Yao, Chuanle Li, Wei Wang, Yupei Yang * and Bin Li *

TCM and Ethnomedicine Innovation & Development International Laboratory, School of Pharmacy, Hunan University of Chinese Medicine, Changsha 410208, China

* Correspondence: yangyupei24@163.com (Y.Y.); libin@hnucm.edu.cn (B.L.)

Table S1 Liver Pathology Scoring System (Ishak Scoring System)

Liver histopathology	Scoring	Specific description of liver status
Hepatocyte necrosis	0 points	No necrosis
	1 point	Less than 50% necrosis
	2 points	50% - 75% necrosis
	3 points	More than 75% necrosis
Degree of fibrosis	0 points	No fibrosis
	1 point	Fibrosis is present but not severe
	2 points	Mild fibrosis, characterized by reticular fibrosis and portal fibrosis
	3 points	Moderate fibrosis, characterized by reticular and obvious coarse fibrosis within the lobules
	4 points	Severe fibrosis, with marked bridging fibrosis and possible cirrhosis formation
Degree of inflammation	0 points	No inflammatory cell infiltration
	1 points	Mild inflammatory cell infiltration (less than 2 inflammatory cells per 20x magnification field)
	2 points	Moderate inflammatory cell infiltration (2 - 4 inflammatory cells per 20x magnification field)
	3 points	Severe inflammatory cell infiltration (more than 4 inflammatory cells per 20x magnification field)
Biliary tract injury	0 points	No biliary tract injury
	1 point	Mild biliary tract injury
	2 points	Obvious biliary tract injury

Table S2 Pathological scores of the liver in each group

	Hepatocyte necrosis scoring	Fibrosis scoring	Inflammation scoring	Biliary tract injury scoring
**Control**	0 points	0 points	0 points	0 points
**Model**	2 points	0 points	2 points	0 points
**Silymarin**	1 points	0 points	1 points	0 points
**L-KCR**	2 points	0 points	2 points	0 points
**M-KCR**	1 points	0 points	1 points	0 points
**H-KCR**	1 points	0 points	1 points	0 points
